# Correction: LGBTQ+ identities in the Indian audiovisual advertisements: A content analysis

**DOI:** 10.1371/journal.pone.0318801

**Published:** 2025-01-30

**Authors:** The PLOS ONE Staff

## Notice of republication

This article was republished on January 16, 2025, to correct an error in the author list: Khuman Bhagirath Jetubhai was erroneously displayed as Bhagirath Jetubhai Khuman. The publisher apologizes for the error. Please download this article again to view the correct version. The originally published, uncorrected article and the republished, corrected article are provided here for reference.

## Supporting information

S1 File Originally published, uncorrected article. (PDF)

S2 File Republished, corrected article. (PDF)
