# Supplementary material for: LGBTQ+ identities in the Indian audiovisual advertisements: A content analysis
Source: PLoS One. 2024 Jan 18;19(1):e0294071. doi: 10.1371/journal.pone.0294071 (PMC10796037; doi:10.1371/journal.pone.0294071)
Supplement: Attachment — Submitted filename: LGBTQ Paper review.docx [file pone.0294071.s001.docx]

Hi,

First of all, many congratulations to the author for working on such an important topic. Although, one can notice author’s hard work here but this paper still lacks scientific rigor. I have a few following suggestions to improve the quality of this paper:

1. Comments:
2. URL of the data is not working
3. Take professional help for editing.
4. Under introduction, my suggestion to the authors would be add a few more studies linking advertisement with identity development among in-general population as well as among minority group of sexual preference.
5. What is LGBTQ+^2^ ? (Written in 3^rd^ para of intro.)
6. No doubt this paper is based on an important aspect of our society that still needs to be studied by the Indian researchers. However, this paper lacks a strong rationale. It is my advice that the authors should write more convincing and stronger rationale for this study.
7. No need to write importance of this study under introduction section.
8. Under methodology, you have written that 16 web sources were traced for advertisement campaigns. Suggest you to give a reason for choosing these 16 web sources. On what basis these 16 web sources were shortlisted for this study?
9. The title suggest that the paper is based on a content analysis but except title of the paper there is no mention or description how the author have used this method for analysis.
10. Did you follow any criteria w.r.t. timeline of advertisement or language (as India is a country of many languages).
11. Your statement, “Thus, 105 observations of marketing campaigns were collected and used for the various analyses discussed below:”, is very unclear. What do you mean by ‘various analyses? As per my understanding, you are referring to various coding categories. Further, it is again unclear, whether these are inductive or deductive categories and on what basis you have decided on these so-called ‘various analyses?
12. Why did you analyse advertisements released yearly and monthly? You need to justify that too.
13. Further, you should have first developed research questions and hypothesis (you can find many good papers on content analysis that have developed hypothesis) to justify the requirement of your ‘various analyses’ mentioned under methodology.
14. Findings of this study is interesting but it lacks scientific rigour.
15. Suggest you to not use bullet points for writing your discussion.
16. Under 1^st^ point of discussion, please correct the citation of (Group M, 2022).
