# Supplementary material for: LGBTQ+ identities in the Indian audiovisual advertisements: A content analysis
Source: PLoS One. 2024 Jan 18;19(1):e0294071. doi: 10.1371/journal.pone.0294071 (PMC10796037; doi:10.1371/journal.pone.0294071)
Supplement: Attachment — Submitted filename: rebuttal Letter (2).docx [file pone.0294071.s002.docx]

**Rebuttal Letter**

2. URL of the data is not working.

**Response:** All URLs listed in the Reference section have been verified, and any broken links have been replaced with working URLs.

3. Take professional help for editing.

**Response:** The article has been edited and proofread in accordance with the suggestions provided by the reviewer.

4. Under introduction, my suggestion to the authors would be add a few more studies linking advertisement with identity development among in-general population as well as among minority group of sexual preference.

**Response:** This suggestion has been implemented, and additional studies discussing the link between advertisement and identity development have been included in the introduction section.

5. What is LGBTQ+2 ? (Written in 3rd para of intro.)

**Response:** The superscript "2," which referred to an endnote number, has been merged with the main text since the journal guidelines do not permit separate food notes or endnotes.

6. No doubt this paper is based on an important aspect of our society that still needs to be studied by the Indian researchers. However, this paper lacks a strong rationale. It is my advice that the authors should write more convincing and stronger rationale for this study.

**Response:** In accordance with the reviewer's feedback, the rationale of the study has been included in the introduction section.

7. No need to write importance of this study under introduction section.

**Response:** The guidelines from PLOS ONE recommend incorporating the significance of this study within the introduction.

8. Under methodology, you have written that 16 web sources were traced for advertisement campaigns. Suggest you to give a reason for choosing these 16 web sources. On what basis these 16 web sources were shortlisted for this study?

**Response:** The justification for selecting these 16 web sources has been included in the methodology section.

9. The title suggest that the paper is based on a content analysis but except title of the paper there is no mention or description how the author have used this method for analysis.

**Response:** An explanation of the content analysis method has been included in the methodology section for clarity and comprehension. The same section contains seven bullet points that outline the application of content analysis in the paper.

10. Did you follow any criteria w.r.t. timeline of advertisement or language (as India is a country of many languages).

**Response:** All advertisements, regardless of language and release time, have been selected, and these criteria are now explicitly stated in the methodology section.

11. Your statement, “Thus, 105 observations of marketing campaigns were collected and used for the various analyses discussed below:”, is very unclear. What do you mean by ‘various analyses? As per my understanding, you are referring to various coding categories. Further, it is again unclear, whether these are inductive or deductive categories and on what basis you have decided on these so-called ‘various analyses?

**Response:** To eliminate any ambiguity, I have included an explanation of the fundamental content analysis process. The selection criteria for these coding categories have already been outlined in the methodology section.

12. Why did you analyses advertisements released yearly and monthly? You need to justify that too.

**Response:** In the methodology section, we provide the rationale for categorising advertisement release data into yearly and monthly criteria. To repeat, this categorisation serves the purpose of identifying which specific month of the year sees the highest volume of advertisements released. Additionally, it helps us understand the reasons behind variations in advertisement releases, such as higher numbers during Pride Month or lower numbers during non-Pride months. Moreover, it allows us to investigate the increased number of advertisements released after 2018, which can be attributed to the decriminalisation of Section 377.

13. Further, you should have first developed research questions and hypothesis (you can find many good papers on content analysis that have developed hypothesis) to justify the requirement of your ‘various analyses’ mentioned under methodology.

**Response:** Research questions and hypothesis have been incorporated.

14. Findings of this study is interesting but it lacks scientific rigour.

**Response:** In this study, content analysis is employed to quantify the occurrence of specific advertisement types or those featuring particular characters. The resulting counts are then interpreted through basic statistical techniques, such as correlation, percentages, and summation, using Microsoft Excel software. Notably, this paper does not employ advanced or intricate statistical methods to derive its findings. This may have led the reviewers to question its scientific rigour. Nevertheless, it's important to note that content analysis is a well-established research methodology, and like any other approach, it has its limitations, which are discussed in the research paper's limitations section.

15. Suggest you to not use bullet points for writing your discussion.

**Response:** Bullet points have been replaced with subsection headings.

16. Under 1st point of discussion, please correct the citation of (Group M, 2022).

**Response:** The working URL has been inserted.
